# Videos

**DOI:** 10.1155/1995/31350

**Published:** 1995


### V001

### CENTRAL HEPATIC RESECTION WITH TOTAL CAUDATE LOBECTOMY FOR CARCINOMA OF THE HEPATIC HILUS UNDER PARTIAL LIVER OCCLUSION.

J. Belghiti,R Noun.
Department of Digestive Surgery, Hôpital Beaujon, F - 92110 Clichy

A 43 years old female with carcinoma of the hepatic hilus underwent 6 weeks preoperative transhepatic biliary drainage. Arteriography demonstrated the patency of both arterial and portal branches. After improving jaundice a "en bloc" resection was performed including common bile duct, lymphadenectomy and liver segments I, IV and V. Resection was performed under partial liver exclusion including portal triad clamping and occlusion of both middle and left hepatic veins.
Central biliary resection resulted in 3 tumor free central bile ducts. For reconstruction a 50 cm jejunal segment was used with end-to-side cholangiojejunostomy.
No postoperative complication occurred and she was discharged from the hospital on the 17$^{th}$ postoperative day. She is free of recurrence on e year later.

### V002

### LEFT HEPATECTOMY WITHOUT VASCULAR OCCLUSION FOR LIVING RELATED TRANSPLANTATION.

J. Belghiti, R. Noun, Y. Révillon*, D. Jan*
Departments of Digestive Surgery, Hôpital Beaujon, F - 92110 Clichy & *Department of Pediatric Surgery, Hôpital Necker, F - 75015 Paris.

A 30 years old man underwent a left lateral segmentectomy as a graft-harvesting for transplantation in his 3 years old daughter with biliary atresia.
Donor liver volumetry using CT scan showed a left lobe of 300 cc. Anatomic analysis of the hepatic artery using arteriography demonstrated a common branche of segments II, III and IV. Anatomic analysis of hepatic veins was obtained by ultrasonography.
Donor operation: intraoperative US scan confirmed the presence of a common trunk of the middle and left hepatic veins. The left hepatic artery was exposed near its origin from the proper hepatic artery and the left portal vein was exposed posteriorly near the caudate lobe. The left triangular and hepatogastric ligaments were dissected from the liver and the common trunk of the middle and left hepatic veins was isolated.The hepatic parenchyma was transected using ultrasonic dissector without blood vessel clamping. After parenchymal transection from the right lobe and from the anterior part of the caudate lobe, the left bile duct was transected. When the liver was free on its hepatic artery, portal vein and hepatic vein which were then transected, the graft was flushed in situ through the left portal vein first with 200 mL of Ringer's solution. On the back table the liver was perfused with 1000 mL UW solution during hepatic veins reconstruction. Fibrin glue (®Tissucol, Immunotransfusion) was applied on the cut surface of the liver for secure hemostasis. Blood loss was estimated to 800 mL for which autotransfusion was performed.
Postoperative course of both donor and recipient were uneventful and they were discharged from the hospital respectively on the 7 and 21 postoperative days.

### V003

### LIVER RESECTION: IMPROVED SEGMENTAL RESECTION OF THE RIGHT LIVER - THE POSTERIOR INTRAHEPATIC GLISSONIAN APPROACH

B. Launois, GG. Jamieson, J. Terblanche
Department of Digestive Surgery and Transplant Unit, Hopital Pontchaillou, CHU Rennes, 35033, France

A new technique, which has simplified segmental liver resection, is described. The individual Glissonian sheaths supplying the segments of the right liver are approached by posterior intrahepatic dissection from the porta hepatis. The segment to be removed is clearly delineated by clamping individual sheaths which produces a colour change. This permits accurate resection of a single liver segment.

### V004

### HEPATIC RESECTIONS ARE SAFE: A CONSECUTIVE SERIES OF 100 HEPATECTOMIES.

M.MORINO,C.MIGLIETTA,C.GARRONE,V.FESTA,M.FORNARI.

CLINICA CHIRURGICA I.UNIVERSITY OF TURIN.ITALY.

In the last decade liver resection has become an established treatment for benign and malignant diseases.Currently the operative mortality for non cirrhotic livers is 5% and for cirrhotic livers 15 %.From October 1990 to November 1994 100 hepatic resections were performed consecutevely.58 patients were operated for liver metastases (with 10 rehepatectomy for recurrent disease).In this group of patients 52 had metastases from colorectal carcinoma, 2 from gastric cancer ,2 from breast tumor;1 patient from pulmonary carcinoid and 1 from a caval leyomiosarcoma. 22 liver resections were realized for hepatocellular carcinoma (16 on cirrhotic liver);8 patients were resected for gallbladder carcinoma,1 for choledocal carcinoma and 1 for cholangiocarcinoma.2 hepatectomies have been performed for intrahepatic lithiasis,2 adenomas,2 focal nodular hyperplasia,1 hydatic cyst and 3 hemangiomas.71 minor and 29 major hepatectomies were realised.There were no deaths on 84 resections in non-cirrhotic patients, while 1 cirrhotic patient died from hepatic postoperative insufficiency.The mean hospital stay was 8 days for non cirrhotic livers and 10 days for cirrhotic patients.Morbidity was respectively 13 and 33%.In conclusion improvements in operative and anesthetique techniques,a better anatomic understanding and an earlier detection of hepatic neoplasm have improved the safety of hepatectomies.

### V005

**RESECTION OF THE CAUDATE LOBE OF THE LIVER**

Gozzetti G., Mazziotti A., Jovine E., Grazi G.L., Principe A., Morganti M., Pierangeli F.
2°Department of Surgery, Policlinico S.Orsola, University of Bologna, Italy

The video shows the surgical anatomy of the caudate lobe by means of three dimensional computerized imaging.
The caudate lobe has an indipendent vascularization originating from both right and left main glissonian pedicles; the latter are predominant. Caudate lobe resections can be associated either to a right or left hepatectomy, or to a resection of 4th segment or right posterior segments (7 cases in our experience, mostly for Klatskin tumors). Isolated resections of the caudate lobe are technically more difficult. Benign tumors (4 cases), hepatocellular carcinomas with cirrhosis (3 cases), and in 1 case a metastatic lesion were the indications to this type of surgery.
The video presents an isolated caudate lobe resection for liver cell adenoma.

### V006

THE PANCREATIC HEAD PARTIAL RESSECTION ASSOCIATED TO LONGITUDINAL PANCREATOJEJUNOSTOMY IN CHRONIC PANCREATITIS - EXPERIENCE WITH 30 PATIENTS

F. Callejas Neto, J.C. Pareja, E.A. Chaim, V.F. Pilla
Department of Digestive Diseases Surgery, State University of Campinas, Campinas-SP, Brazil

The longitudinal pancreatojejunostomy as described by Partington and Rochelle is acepted as the standard procedure in patients with chronic pancreatitis associated with ductal dilatation. However in the group presenting with severe pancreatic head complications the pylorus preserving Whipple represents a more widespread procedure. The pancreatic head partial ressection associated to pancreatojejunostomy as proposed by Frey in 1987 represents an alternative to ressective procedures.
From 1990 to 1994 30 patients were submited to pancreatic head partial ressection associated to pancreatojejunostomy. All patients were men. The mean age was 39,8 years (range 26-56 years). The indications to operate were severe pain (100%), biliary obstruction (76,6%), Pancreatic cysts (59,9%) duodenal obstruction (13,3%) wirsunghorragia (6,6%) and persistente of pain after longitudinal pancreatojejunostomy (3,3%). Intrapancreatic biliary decompression was performed in 23 patients (76,6%). The pre and post-operative investigations included: pain, body weight, bioquemical tests (alkaline phosphatasis, glucose, fecal fat) CT, ultrassonography, ERCP, hospital readmissions. The follow-up average was 36 months. There was no operative mortality. All but one patient were free of pain, the mean weight gain 9 kg (range 2-34 kg). Two patients with elevated alkaline phosphatasis and were submited to endoscopic sphincterotomy, one patient with acute cholecystitis, and one patient with moderate residual pain.
The pancreatic head partial ressection associated to longidutinal pancreatojejunostomy is a safe procedure, with excelent postoperative outcome in regard to clinical as well as metabolic data, in patients with severe pancreatic head complications in chronic pancreatitis.

### V007

PYLORIC PRESERVING PANCREATICODUODENECTOMY (PPPD) FOR CHRONIC PANCREATITIS

RL Rossi, J Buyske, R Martin
Department of General Surgery, Lahey Clinic, 41 Mall Road, Burlington,MA 01805

A step wise description of pyloric preserving pancreaticoduodenectomy is presented using colored art work and video. Technical highlights include preservation of the blood supply to the pyloris, dissection of the neck of the pancreas in the presence of inflammation, and techniques for vascular control. Our experience with over 45 PPPDs for pancreatitis is summarized. Potential benefits include improved gastric emptying, better postoperative weight gain, and better pain control.

### V008

PANCREATICODUODENECTOMY FOR AMPULLARY CARCINOMA - SPECIAL FOCUS IN DUCT-TO-MUCOSA PANCREATOJEJUNOSTOMY

J.C. Pareja, F. Callejas Neto, V.F. Pilla
Department of Digestive Diseases Surgery, State University of Campinas, Campinas-SP, Brazil

This video shows the technical aspects of the pancreaticoduodenectomy in an ampullary carcinoma regarding the limphadenectomy (R2), apropriate anatomic vascular identification and meticulous performance of pancreatojejunostomy with duct-to-mucosa anastomosis. In this particular case a lost catether was used through the anastomosis. There was no blood transfusion and the procedure lenght was 5 hours. The authors put emphasis in the safety of the procedure in terms of morbidity and mortality.

### V009

**AMPULLARY ADENOMYOMA PLAYING THE PART OF PANCREATIC CANCER**

E.C. Tsimoyiannis, E. Lekkas, A. Tassis, G. Floras, S. Stefanaki-Nikou*
Departments of Surgery and Pathology*, G. Hatzikosta General Hospital, Ioannina, Greece.

Benign tumors of the extrahepatic bile ducts are rare and adenomyoma of the ampulla of Vater is extremely rare. We present a video tape of a 56-year old man who came to the hospital for jaundice, (TBL=26 mg/dl, DBL=20 mg/dl), weight loss, anorexia, dark urine, light stools and palpable gallbladder. CT scan detected tumor of the head of pancreas 3 cm in diameter. ERCP and preoperative biliary decompression were unsuccessful. The preoperative diagnosis was tumor of the head of the pancreas probably carcinoma. On the operative table a resectable mass of the head of the pancreas was found. The tru-cut needle biopsy showed chronic pancreatitis. To correct the jaundice, liver function and nutritional deficiency the first step of operation was a cholecystojejunostomy. Twenty days later the improvement of general condition of the patient was evident and a Whipple pancreaticoduodenectomy was performed. Examination of the excised specimen revealed a mass in the head of the pancreas and complete obstruction of common bile and pancreatic ducts at the level of the ampulla. Histological examination revealed adenomyoma of the ampulla of Vater, complete obstruction of the ducts and chronic pancreatitis of the head of the pancreas. The patient s discharge was on the 9th postoperative day in excellent condition.

### V010

ADJUVANT INTRAOPERATIVE RADIATION THERAPY (IORT) AND REGIONAL PANCREATECTOMY

E. Bodner, K. Glaser, A. Klingler
Second Department of Surgery, University Hospital of Innsbruck, Austria

45 patients received adjuvant IORT during duodenopancreatectomy, 33 of them suffering from ductal carcinoma. In 22 comparable cases the operation was done without IORT. The median overall survival is 21 months. Although we cannot define the prognostic influence of IORT so far, the results altogether are remarkable. The video shows the strategy as well as the operative procedure of this combined therapy step for step including the technique of extended regional lymphadenectomy during Whipple operation.

### V011

LIVER TRANSPLANTATION WITH TEMPORANEOUS PORTO-CAVA SHUNT

V.Mazzaferro, A.Pulvirenti, E.Regalia, F.Bozzetti, R.Doci, L.Gennari.
Liver Unit, National Cancer Institute, Milan, Italy

Standard technique of Orthotopic Liver Transplantation (OLT) requires extracorporeal circulation (external veno-venous by-pass) during the anhepatic phase of the operation. In recent years several techniques for avoidance of ext.v-v by-pass have been proposed. Our experience with temporaneous end-to-side porta-caval shunt during the anhepatic phase of OLT is reviewed in a twenty minutes video with the assistance of computer graphics.
Porta-caval shunt (internal by-pass) if associated with piggy-back caval reconstruction at the time of engraftement could avoid ext. v-v by-pass and significantly reduce both the anhepatic phase and the total operation time.
In our experience use of porta-caval shunt and piggy-back reconstruction completely replaced ext. v-v by-pass in the intraoperative management of liver transplantation in patients with patent portal vein.

### V012

**LIVER TRASPLANTATION WITH PRESERVATION OF RETROHEPATIC RECIPIENT VENA CAVA .**

Gozzetti G., Mazziotti A., Grazi G.L., Jovine E., Gallucci A., Ercolani G.
2° Department of Surgery, Policlinico Sant'Orsola, University of Bologna, Italy.

The video shows the technique of orthotopic liver transplantation with the preservation of the recipient retrohepatic vena cava. This technique allows the maintenance of the caval flow during the anhepatic phase and the avoidance of the veno-venous by-pass. The liver is detached completely from the retrohepatic vena cava with the legation of accessory veins. The main hepatic veins are clamped and the liver removed. In the case shown, an end to side porto-caval shunt has been performed to avoid splancnic congestion during the anhepatic phase. This temporary porto-caval shunt has not been performed in 77% of the cases, without any hemodynamic drawnback. The upper vena cava of the graft is anastomized end to side with the recipient vena cava or preferably end to end with the stump of the middle-left hepatic vein. The lower vena cava of the graft is stapled and the portal vein anastomosis is performed. The piggy back technique has been performed in 7% of our liver transplantations: it is advisable in the case of small grafts, such as reduced size liver grafts, in the presence of previously operations to reduce bleeding of retrohepatic space and when the patient has a prior porto-caval or a meso-caval shunt. A particular indication is fulminant hepatitis, when it can be employed as a bridge procedure before the implant of the graft. A further advantage is economical, since the veno-venous by-pass is not necessary.

### V013

### THORACOPRHENOLAPAROTOMY FOR SUPERIOR EDGE OF 10$^{TH}$ RIB IN THE TREATMENT OF HEPATIC HYDATIDOSIS

S.Mallagray[*], T.Butrón[**], M.Diaz Tie[**], M.J. Castillo[**]
[*]Department of General Surgery Cruz Roja Hospital. Madrid.
[**]I Department of General and Digestive Surgery. 12 de Octubre Hospital. Madrid. Spain.

Hepatic hydatic cyst are located more frecuently in the right lobe, being in more than 60% of the cases in posterosuperior segments. Radical surgery is the most effective. We proposed the thoracophrenolaparotomy for superior edge of 10$^{th}$ rib (TPL10) how suitable surgical incision for hydatic cysts of that location.
MATERIAL AND METHODS: Supported for the results of a prospective study of 48 patients operated on wiht this incision we show two cases of hepatic hydatic cysts treated surgically with a close total pericystectomy and an open total pericystectomy by a TPL10 incision.
RESULTS: With TPL10 we had a large operative field performing radical techniques in all patients. We used a modified trocar of Dermileau attached to a negative pressure suction apparatus that allowed a sudden aspiration of the cyst in the open pericystectomy. After we performed total pericystectomy and commun duct exploration if there was biliary fistula. Combined techniques by that incision were done in 8 patients. Morbidity was 12.5%.
CONCLUSIONS: TPL10 allowed perform radical techniques in all cases with both low morbidity and postoperative hospital stay. It was a good incision to perform combined techniques. TPL10 seems to be a suitable incision for posterosuperior hepatic hydatic cysts, especially those of large size.

### V014

### LIVER TRANSPLANTATION

E.Hadjiyannakis, R.Iakoumenta, S.Drakopoulos, N.Georgopoulos, A.Poultsidi, N.Nikitakis, S.Mathioulakis, H.Harisis, F.Filippidou, E.Plessia.

1st Surgical Department and Transplant - "Evagelismos" Hospital of Athens.

Liver transplantation has become the cornestone of treatment of end-stage hepatic failure.
Concerning indications of liver transplantation the commonest are: livercirrhosis due to viruses (HBsAg), diseases of metabolism and primary hepatic tumors,
Main stages of liver transplantation are:
1. Hepatectomy of the cadaveric graft.
2. Hepatectomy of the recipient.
3. Veno-venous by pass.

Revascularization of hepatic graft includes:Anastomosis of a. S.V.C.V.

b. P.V.

c. Hepatic artery d. I.V.C.V.

e. end to end bike duct anastomosis.

### V015

### RADICAL TREATMENT OF LIVER HYDATIDOSIS

M. A. Secchi
Surgical Division "B". Hospital Italiano of Rosario. Argentina.

Video/Pal-N/12 minutes

**PATIENT**
27-year-old white woman, married with 3 children.
Symptoms: epigastric and right hypocondric pain
Signs: 8 cm-rounded palpable tumor in epigastrium,elastic consistency
Antecedents: positives focus for hydatidosis 20 years earlier, normal serology
Echography and CT Scan: Cyst tumor in Segment III of the liver with hydatidic aspect. Gallbladder lithiasis and acute cholecystitis

**METHOD (SURGERY)**
Bisubcostal anchor like incision.
Standar cholecystectomy.
Cysto-gastrolysis.
Segment III resection around the cyst.
Hemostatic suture.
Fibrine solution in site section.
Normal intraoperative cholangiography.
Blood loss estimated in 100 ml.

**RESULT**
There were no post-operative complications, and the six months follow-up was normal.

**CONCLUSION:** As was just seen in the surgery, this is an effective procedure when the operative risk is low.

### V016

### LAPAROSCOPIC DISTAL PANCREATECTOMY WITH SPLEEN PRESERVATION FOR CYSTADENOMA .

A. Paganini, F. Feliciotti, M. Guerrieri, E. Lezoche
Istituto di Scienze Chirurgiche. Università di Ancona, Italy.

The purpose is to report on the laparoscopic treatment of an asymptomatic benign cystic neoplasm of the body and tail of the pancreas occurring in a 20 years old, professional athletic, female patient who was treated by laparoscopic corpo-caudal resection of the pancreas with preservation of the spleen.
The cystic neoplasm, measuring at CT scan 10 x 10 cm. in size, extended from the level of the celiac trunk to the level of the inferior pole of the left kidney. Serum CEA and Ca 19-9 were negative. 3 trocars were used, one at the umbilicus and two in the left and right hypochondria, respectively. The gastro-colic ligament was opened, and the body and tail of the pancreas including the lesion were carefully dissected from the splenic vein and artery. A plane behind the body of the pancreas was bluntly dissected free to apply a 65 mm. linear stapler (3.85 mm staples) for stapling and resection at this level with adequate margins of apparently normal tissue. Two 35 mm linear staplers (2.5 mm staples) were applied to divide the tail of the pancreas from the spleen. The specimen was introduced inside a specimen-retrieval bag and the cyst was puncture-drained avoiding to spill material outside the bag. After specimen removal, intraoperative cytological fluid analysis and frozen section of the cyst allowed to reasonably exclude the malignant nature of the tumor. Fibrin glue was applied to seal the pancreatic suture line and two drains were employed.
No complications were observed in the postoperative course. Drains were removed after 3 and 6 days, respectively. The patient was dismissed after 9 days to resume her athletic activity. Histology confirmed a benign mucinous cystadenoma.
The expected benefits of laparoscopic surgery were observed in this young, female patient. Laparoscopic spleen preserving distal pancreatectomy should be considered the treatment of choice for benign corpo-caudal pancreatic lesions in the hands of skilled surgeons with specific laparoscopic operative experience of the pancreatic anatomy.

### V017

**PANCREATIC SEUDOCYST TREATED BY LAPAROSCOPIC INTRALUMINAL STAPLED CYSTOGASTROSTOMY.** M Trías, E.Mª Targarona, C Balagué, A Cifuentes, J Martinez, J Tabet. Service of Surgery, Hospital Clínic, University of Barcelona. Barcelona. SPAIN.

The standard treatment for pancreatic pseudocyst is digestive diversion. Recently, laparoscopic intraluminal cystogastrostomy has been described, and a window is created between the cyst and the stomach wall, but morbidity after cystogastrostomy arises in bleeding of the cut surface. We present the technique for stapled intraluminal cysto-gastrostomy, that offers a similar treatment to the open way with a laparoscopic approach. **Case report:** A 29 y. old man developed a 6 cm pseudocyst located in the body of the pancreas after a biliary acute pancreatitis. Laparoscopic cholecystectomy with intraoperative cholangiography was performed. 4 months later, a control CT scan showed the persistence of the image and surgical treatment was proposed. **SurgicalTechnique:** After creation of the pneumoperitoneum, the abdominal exploration showed a mass that displaced the gastric body. Two 12 mm trocars with a balloon were inserted into the gastric cavity through the fundus and antrum an the balloon were inflated. The gastric wall was pulled out to the abdominal wall and the stomach was distended with air. Another camera was inserted into de gastric cavity and a flexible tip ecoendosonography probe permitted to choose the more adequate place for the cysto-gastrostomy. The cyst was punctured and the orifice was enlarged. Then, an endostapler device was inserted through the hole and fired and a wide cystogastrostomy was created. After the retrieval of the trocars, both gastric holes were closed with an endo-stapler The patient returned to oral feeding at 48 h. and was discharged at 7 d. **CONCLUSION:** Refinement of laparoscopic and intraluminal surgical techniques offers a minimal access surgery for treatment of selected pancreatic pseudocysts.

### V018

LAPAROSCOPIC TREATMENT OF NON PARASYTIC LIVER CYSTS WITH OMENTAL TRANSPOSITION FLAP

C. Zornig, A. Emmermann, X. Rogiers, Ch. E. Broelsch
Dept. of General Surgery, University Hospital Hamburg, Germany

Surgical therapy of non parasytic liver cysts is only indicated in case of clinical symptoms or continuously increasing volume. As laparoscopic surgery for other indications has shown a benefit concerning postoperative pain, convalescence and cosmetic result, we chose this technique in case of large solitary liver cysts.

From 1991 to November 1994 18 patients underwent a laparoscopic resection of the cystic roof. To avoid recurrence the greater omentum was seperated from the transverse colon and placed into the cystic cavity in the last 13 patients. Follow-up data were required from all patients by repeat ultrasound.

All patients were female and had an average age of 54 (39-73) years. Fifteen were symptomatic and 3 required surgery because of rapid growth. Cysts had a diameter of 10 to 17 cm, volume varied from 800 to 3000 ml. Mean operation time was 100 (50-145) min. Postoperatively bile leakage in 1 patient stopped under drainage with a Sonnenberg-catheter. Hospitalisation time was 2-14 (mean 4) days. Among our first patients without omental transposition there were 2 early relapses (one operated on conventionally, one asymptomatic). After a median follow-up of 19 (1-43) months all other patients are free of recurrence.

According to our experiences the laparoscopic treatment of non parasytic liver cysts is as safe and effective as the conventional one but has the advantages of the minimal invasive approach.

### V019

DIFFERENT OPTIONS IN THE LAPAROSCOPIC MANAGEMENT OF BENIGN PANCREATIC CYSTIC LESIONS.

M.MORINO,C.GARRONE,L.LOCATELLI (*),C.MIGLIETTA.

CLINICA CHIRURGICA I. UNIVERSITY OF TURIN.ITALY.
OSPEDALE EVANGELICO VALDESE ,TURIN.ITALY (*).

The laparoscopic approach has been recently proposed as one of the therapeutic options in the management of benign pancreatic cystic lesions.The video shows 3 different clinical situations:

1) a laparoscopic cysto-gastrostomy and cholecystectomy for a pancreatic pseudocyst in a patient suffering from chronic pancreatitis of biliary origin;
2) a laparoscopic cysto-gastrostomy for a huge pancreatic pseudocyst developped 8 months after a Whipple procedure;
3) a laparoscopic resection of a 5 cm cyst located in the head of the pancreas (uncinate process).

The postoperative course was uneventful in every case with an hospital stay of 4,6 and 3 days respectevly.
We believe that,in the management of pancreatic cystic lesions,the laparoscopic approach is an attractive alternative either to open surgery,or to endoscopic and radiological treatments, provided that any potential malignancy has been fully explored and excluded.

### V020

Laparoscopic Resection of a Large Recurrent Benign Liver Cyst

Michael K W LI
Department of Surgery, Pamela Youde Nethersole Eastern Hospital, HONG KONG.

This ten minute video shows the laparoscopic resection of a large recurrent benign liver cyst.

The patient is a 73 year old female who first presented with an epigastric mass in 1991 to the department of surgery. Subsequent investigations including ultrasound and CT confirmed a 15cm benign left lobe liver cyst and percutaneous aspiration under ultrasound was successfully performed. This patient returned with a recurrent epigastric mass in 1994 and ultrasound confirmed a 15cm recurrent cyst. Laparoscopic guided drainage with marsupilisation was performed. This was followed by a recurrence within 2 months and the cyst measured 18 cm in size. The recurrent cyst was approached laparoscopically again.

The patient was placed in a supine position and 4 trocars were used for the procedure. The largest trocar was 12mm allowing the use of an Endo-GIA. Omental adhesions were taken down and the cyst was opened and clear fluids were drained. The cyst wall was divided with Endo -GIA up to the edge of liver tissues resulting in two thirds of the cyst resected. Omentalplasty was done to the remnant with an Endo stapler. A silicone drain was inserted for drainage which was removed on day 3 postop. Her postoperative recovery was complicated by a minor chest infection which responded to antibiotics therapy. She was discharged home on day 10.

The successful outcome of resecting the cyst laparoscopically has encouraged the application of this technique to further cases.

### V021

### LAPAROSCOPIC CHOLECYSTECTOMY WITH A SINGLE TROCAR FOR BETTER COSMETIC RESULTS IN SELECTED PATIENTS

A. Paganini, *D. Lomanto *M. Nardovino *M. Sottili, E. Lezoche
Istituto di Scienze Chirurgiche, Università di Ancona. *I.N.I. Canistro, L'Aquila. Italy

A technique to perform laparoscopic cholecystectomy (LC) with a single trocar is reported, with the aim of achieving better cosmetic results in selected, highly motivated patients.
Criteria for selection were patients' request for better cosmetic results, female sex, young age, near ideal body weight, absence of scars from previous upper abdominal operations, recent onset of symptoms. With closed pneumoperitoneum created at the umbilicus, a 10 mm. trocar is positioned and a 10 mm. optics is used to explore the peritoneal cavity. A Pneumoneedle with Pixie Microendoscope (Origin Medsystems Inc.) is introduced under vision at the right paramedian epigastrium, lateral to the falciform ligament. The 10 mm optic is removed and LC is performed under microendoscopic vision. Three loop sutures on straight needle are introduced across the abdominal wall below the right costal arch, passed with a needle-holder through the gallbladder wall and exited through the abdominal wall, to raise the gallbladder and provide exposure of Calot's triangle. The cystic artery and cystic duct are bluntly isolated and closed with clips. I.o. cholangiography is performed with an Olsen clamp and 4 Fr. ureteral catheter. After closing the cystic duct stump with clips, the cystic duct and artery are divided, the gallbladder is freed from its liver bed and removed at the umbilicus. Suction/irrigation and closure of the umbilical defect under vision completes the procedure.
This method was attempted and successfully performed in 3 female patients, aged 25,33,37 years, Body Mass Index of 22.1,22.3,22.6, respectively. I.o. cholangiography was employed in 1 case. Operative time was 55,65,70 minutes. No i.o. complications were observed, the postoperative course was uneventful and the patients were dismissed 48 hours later.
Better cosmetic results are obtained by single trocar LC, which appears from this limited experience to be feasible and safe in highly selected patients.

### V022

### Laparoscopic surgery : introdution and training . The role of the teorical and practical courses.

Girao, R. ; Real, A. ; Schiappa, J.M. ;
Hospital Santo Antonio dos Capuchos
Serviço 6 Cirurgia ( Prof. Jorge Girao )

In this video we try to show the general structure of the laparoscopic courses organised by our group . These courses are done for surgeons , anesthesiologists and nurses .

We present several exercises in a organised sequence , starting in simple manipulations in the simulator and finishing in a operation performed on a animal .

In this video are also made considerations about philosophy on this type of preparation and is done a analisi s of the results of 8 courses done untill now .

### V023

### LAPAROSCOPICALLY ASSISTED REPAIR OF BILIO-ENTERIC FISTULAE

**F. ÇELEBİ, D. GÜZEY, E. OKAY, M.A. GÜRSOY, T. ÇÖMEZ, R.KAPLAN.**

*Vakıf Gureba Hospital, II. Dept. of General Surgery, Istanbul, TÜRKİYE*

Since the first performed laparoscopic assisted cholecystectomy procedure by Mouret, laparoscopic surgery has moved at an accelerating pace which has no limit. One of the known contra - indications of laparoscopic cholecystectomy is bilio-enteric fistulae .

With individual cases, trials with new techniques and procedures are constantly attempted in such situations.

This is a report of such a case, 53 years old morbidly obese female was diagnosed with cholelithiasis. Further laparoscopic diagnosis revealed a fistulae in the area between fundus of the gallbladder and hepatic flexura of the colon The procedure was continued laparoscopically and the gallbladder and fistulae were freed by dissection. Gallbladder and fistulae together with the colon was taken out of the abdomen through a small incision. Fistulae was resected and the defect in the colon was repaired using interrupted sutures. The total duration of the procedure lasted two hours and fifteen minutes.

The patient was discharged on the fifth postoperative day without any complication.

Safe and successful laparoscopic treatment of bilio-enteric fistulae is still a controversy though margins of contra-indications need to be constantly forced.

### V024

### LAPAROSCOPIC SUBTOTAL CHOLECYSTECTOMY FOR THE "DIFFICULT" GALLBLADDER

PC Bornman, PJ Gallagher, JEJ Krige, J Terblanche.

*Department of Surgery, University of Cape Town, South Africa.*

Dissection of Calot's triangle followed by a standard cholecystectomy can be hazardous in the presence of severe inflammation of the gallbladder (GB) or the presence of cirrhosis with portal hypertension. In this setting open subtotal cholecystectomy has been proven to be a safe, simple and definitive procedure (1).
This study and the video demonstrate application of this technique to laparoscopic subtotal cholecystectomy (LSC). Over a 9 month period from January - September 1994 227 patients presented for laparoscopic cholecystectomy. Ninety-eight were for acute cholecystitis and 10 of this group had conversion to open cholecystectomy. In 11 patients (7 males, 4 females; median age 56 years (range:33-81)) LSC was performed for acute cholecystitis/empyema (8),severe fibrosis (2) and cirrhosis with portal hypertension (1). Ten of the 11 had significant comorbidity.
The cystic duct and artery were isolated by blunt dissection and when possible clipped before division. When this was not feasible (eg:a thickened inflamed cystic duct) the GB was divided at the junction of the cystic duct with Hartmann's pouch and then tied with an Endoloop(Ethicon). An initial standard dissection of the GB was commenced but when difficulty was encountered the subtotal modification was used. In this situation the GB was entered and the wall was divided along the junction with the liver using diathermy and clips. Thus, a disc of posterior GB wall was left in situ. Gallstone recovery and irrigation was thorough. A portovac drain was inserted in the 8 cases with empyema. Median operation time was 70 minutes (range:45-120). There were no significant intra- or post-operative complications related to the surgery. Five patients were febrile due to a respiratory cause. Median hospital stay was 4 days (range 2-8days).
LSC is a safe, simple and definitive operation for removal of the difficult gallbladder. This avoids the need for open conversion or cholecystostomy particularly in the high risk patient.

1.Bornman PC, Terblanche J. *Surgery* 1985;98:1-6

### V025

### LAPAROSCOPIC MANAGEMENT OF COMMON BILE DUCT STONES

G. Alexiou, P. Antoniou, M. Papadopoulou, G. Sakorafas, C. Avgerinos, M. Papoudos
2nd Surgical Dpt, Air Force General Hospital, Athens, Greece

The management of patients with common bile duct (CBD) stones is under investigation and represents a technical challenge to the surgeon. Nowadays, the treatment of CBD stones involves a number of alternatives.
This study evaluates the results of laparoscopic CBD exploration and describes the different techniques used to perform it in 26 patients with choledocholithiasis discovered among 1152 patients treated by laparoscopic cholecystectomy (October 1990-December 1994). Initially, 40 patients underwent perioperatively ERCP and sphincterotomy (29 preoperatively and 11 postoperatively). Recently, in 23 more patients a number of different endoscopic techniques were used: trancystic choledochoscopic extraction techniques, intraoperative ERCP, choledochotomy with T-tube drainage. There were 3 open CBD explorations. Mean operative time was 110 min.
Laparoscopic approach for CBD stones is attracting growing interest and our early results advocate that is a feasible method with low morbidity and mortality rate.
The video demonstrates the different techniques of laparoscopic management of CBD stones.

### V026

### RESULTS OF LAPAROSCOPIC SURGERY FOR PARAESOPHAGEAL HERNIAS

J. K. Edoga, M.D.
Department of Surgery, Columbia University College of Physicians and Surgeons, Morristown Memorial Hospital, 100 Madison Ave., Morristown, NJ 07960

Eight patients, 5 females and 3 males, were referred for surgery for paraesophageal hernias within a 12-month period.

Satisfactory repair was achieved utilizing video laparoscopic techniques in all 8 cases. Pulmonary embolism occurring 2 weeks after surgery was the only major complication in this series. Hospital stay was brief, and the postoperative recovery swift in all but 1 case. To date, follow-up reveals relief of symptoms in all of the patients who were symptomatic preoperatively.

Paraesophageal hernias are quite rare. Even though they represent approximately 2% of all hiatal hernias discovered radiographically, they are prone to major complications including visceral infarction, perforation, and bleeding, as well as respiratory problems.

In view of the early results seen in the reported cases, we conclude that laparoscopic repair of paraesophageal hernias is safe and effective and should be recommended if the risks of general anesthesia and pneumoperitoneum are not prohibitive.

### V027

### HEPATIC HEMAGIOMAS - TECHNIQUE OF RIGHT EXTENDED HEPATECTOMY.

E.J.Hadjiyannakis, S.Drakopoulos, R.Iakoumenta, N.Georgopoulos, A.Poultsidi, N.Nikitakis, S.Mathioulakis, H.Harisis.

1st Surgical Department and Transplant Unit - "Evagelismos" Hospital of Athens.

In 32 cases of hepatic hemangiomas we operated upon 9 we performed, 4 right extended hepatectomys, one left extended hepatectomy, a left lobectomy and three Segmental ressections.
We present a right extended hepatectomy for a giant hemangioma, 6.500 kgrs.

### V028

### COMBINATION OF HEPATIC RESECTION AND HEPATIC CRYOTHERAPY FOR THE TREATMENT OF SYMPTOMATIC HEPATIC CARCINOID.

SJ Walker & GJ Poston
Department of Surgery, Royal Liverpool University Hospital, Liverpool, U.K.

The symptomatic control of patients with metastatic hepatic carcinoid often poses a difficult management problem. Although many patients benefit from somatostatin analogue therapy, a significant number of patients fail to respond to medical management and may benefit from liver surgery.

The video will demonstrate the combination of surgical resection of a large carcinoid metastasis in the quadrate lobe of the liver with hepatic cryotherapy to a number of other smaller metastases elsewhere in both lobes. The patient originally underwent pneumonectomy for primary bronchogenic carcinoid and subsequently failed to respond to somatostatin therapy. Following resection, she was clinically and biochemically restored to normality. The video will place great emphasis on the techniques of hepatic cryotherapy.

In conclusion, we recommend this approach for the symptomatic control of patients who fail to respond to medical management.

### V029

**STIMULATION OF MEMBRANE RUFFLING AND MOTILITY OF HUMAN CANCER CELLS BY HEPATOCYTE GROWTH FACTOR**

WG Jiang, S Hiscox, MB Hallett, MCA Puntis
University Department of Surgery, University of Wales College of Medicine, Cardiff, UK

Hepatocyte growth factor / scatter factor (HGF/SF) is a promoter of tumour cell motility and invasion. This study determined the effects of HGF/SF on tumour cell membrane ruffling, an early event in tumour cell motility and invasion.

Membrane ruffling of human cancer cells, HT115 and HRT18, was monitored with a time lapse video recorder which revealed a rapid membrane ruffling and formation of microspikes after HGF/SF stimulation. Onset of membrane ruffling occurred as early as 5 minutes and reached maximum in approximately 30 minutes. HGF/SF exerted the biological effects at a wide range of concentrations (2-100ng/ml). These events were also confirmed by scanning electron microscopy. The ruffling indices were 0.23±0.03 in control and 1.1±0.08 with HGF (10ng/ml). This increased ruffling was related to a increased cell motility as visualised by time lapse video and also confirmed by both an increased phagokinetics in a colloidal gold phagokinetic assay and an increased dissociation from carrier beads in a Cytodex-2 dissociation assays (dissociation indices 1.0±0.1 in control and 6.1±0.3 with HGF/SF).

It is concluded that HGF/SF promotes tumour cell membrane ruffling which is related to a increased motility.

### V030

SINGLE LOBE CAROLI'S DISEASE TREATED BY RESECTION.

A.M.Isla,J.Rouco,F.Rey,L.Cerrada,J.Mosquera.
Dpt. of Surgery, Hospital POVISA,Vigo Spain.

Caroli's disease or congenital dilatation of the intrahepatic bile ducts is a rare biliary disease.

Less than 20% cases (34 cases published up to 1989) are of monolobar type.Calculi are present in 70% of them.Thirty per cent of patients present with recurrent abdominal pain Preoperative diagnosis is seldom made, that, makes adequate management difficult.Definitive therapy, in the monolobar disease,is removal of affected segments.

In the video we present the case of a 66 y/o white woman who, during a routine analysis,was discover to have a raised serum alkaline phosphatase.US and CT examination showed dilatation of bile ducts filled with stones,in the left liver.ERCP failed to outline the intrahepatic ducts.PTC confirmedthe involvement of the left lateral segment.We decide to perform left lateral segmentectomy(Couinaud:left lobectomy).In the film we show the surgical technique, with special emphasis in the left bile duct disection,and caudate lobe removal(to achieve a better approach to distal parts of this conduct) in order to ease the emptiying of calculi, prior the resection.

Two years after the operation the patient is asymptomatic.

We consider the resection as the treatment of choice in the management of localized Caroli's disease in the good risk patient.

### V031

### Intraoperative Sonography of the Liver

K.T.E. Beckurts, A.H. Hölscher, J.R. Siewert

Department of Surgery

Technische Universität Müchen, Germany

In recent years, sonographic techniques have gained great importance for diagnosis and treatment of surgical patients. Besides conventional percutaneous and intraluminal methods, intraoperative techniques have been developed. The sensitivity of intraoperative sonography of the liver has been shown to be superior to that of preoperative CT-scans in the detection of intrahepatic metastases. Extended hepatic resections or resections of intrahepatic tumors with close relationship to central hepatic structures can be facilitated with the help of sonography. Operative laparoscopy is gaining importance in the pretherapeutic staging of malignant diseases, and laparosopic sonography offers additional information and allows for guided biopsy of intrahepatic lesisons of uncertain dignity even for tumors of small size and unfavourable location. Bleeding complications following biopsy can be avoided by controlled compression and coagulation.
In our 12 minute video presentation, we demonstrate the available techniques and indications for intraoperative sonography including colour duplex imaging, laparoscopic sonography and biopsy of intrahepatic lesions guided by laparoscopic sonography. Normal anatomy is demonstrated as well as various examples of typical pathologic findings. The possible benefits and limitations of intraoperative sonography are discussed.

### V032

**SURGICAL THERAPY OF BUDD-CHIARI SYNDROME. A CASE REPORT**

**Forni E., Meriggi F., Morone G.**
University of Pavia (Italy), General Surgical Clinic, IRCCS San Matteo Hospital, Hepato-biliary Surgical Unit

Budd-Chiari syndrome (BCS) is a group of disorders caused by obstruction of hepatic venous outflow. Patients with BCS may be treated by portosystemic decompression or orthotopic liver transplantation.
The case is reported of a young man (age 28 years) with an atypical chronic myeloproliferative disorder characterized by a high thrombotic risk and a slow disease progression. The haematologic disease was associated with a complete hepatic venous thrombosis (BCS). The patient had hepatomegaly, ascites and abnormal liver function (PT < 30%). Encephalopathy was absent. Antithrombin III, protein C and protein S were normal. A biopsy specimen showed intense hepatic congestion and necrosis. Doppler ultrasonography, angiography, CT scan and NMR of the abdomen demonstrated a patent portal vein. The hepatic venous thrombosis involved the suprahepatic inferior vena cava incompletely.
Based on the absence of encephalopathy, the myeloproliferative aetiology of the disease, and the refractory ascites a double prosthetic side-to-side portal-caval and caval-atrial by-pass was devised to decompress the hypertensive IVC and shunt the entire portal venous flow.
The operation was performed through a bilateral subcostal laparotomy and a median sternotomy. With a 10-mm ring-reinforced polytetrafluoroethylene (Gore-Tex) graft a side-to-side portal-caval (suprarenal) shunt was performed. With a 16-mm ring-reinforced Gore-Tex graft a side-to-side caval (infrarenal)-atrial shunt was performed. Before shunting the IVC pressure was 22 cm of saline and the portal pressure was 33 cm. After shunting the ICV pressure was 5 cm of saline and the portal pressure was 15 cm.
The patient survived the operation and the postoperative course was uneventful. Repeated Doppler ultrasonographies demonstrated patency and good function of both the grafts. Permanent anticoagulation therapy with low-dose acetylsalicylic acid is used in the patient who is alive to date and free of ascites and encephalopathy.

### V033

INSULINOMA - TECHNIQUE OF DISTAL PANCREATECTOMY.

E.J.Hadjiyannakis, S.Drakopoulos, N.Georgopoulos, A.Poultsidi, S.Mathioulakis, N.Nikitakis, H.Harisis, M.Mitrakou, S.Raptis.

1st Surgical Department and Transplant Unit - "Evagelismos" Hospital of Athens.

Depending on the location of the insulinoma.
Surgical treatment includes:
- enucleation or resection of the tumor.
- proximal (whipple) pancreatectomy.
- distal pancreatectomy.

We present a case of insulinoma where distal pancreatectomy was performed.

### V034

RESTAURATION OF HEPATICOCHOLEDOCHUS WITH FREE AUTOVENOUS GRAFT

M.Stojanović,M.Jeremić,P.Stojanović, M.Stojiljković,M.Djordjević,A.Bogićević M.Pešić,Z.Cvetković

Surgical clinic and Veterinary station-Niš Serbia,Yugoslavia

This study was performed to asses succes of restauration of hepaticocholedochus(HCH) with free autovenous graft(FAG).In 15 dogs the authors performed a resection of the supraduodenal HCH and arising defect was bridged over FAG-exterior jugular vein,with proximal and distal anastomoses end to end. Fifth months living rate was 55% and most ussualy complication was leakage of anastomoses (22%) and necrosis and perforation of the FAG (11%).The animals were folowed-up 15o days with the control of holestasis and hepatocellurary necrosis parameters.All values were increased in the first postoperative days with normalisation during forth week.Histological examinations performed every 7 days showed adequate vitality of the FAG,with high procent complete epitelisation (33%),but intensive fibrous reaction in some cases.Intraoperative transpapillary cholangiography after 15o days showed absolute funcionality of FAG in 33%,high-procent stenosis in 11% and total obstruction in 11%.Restaurative operations HCH have many theorical advantages compared with biliodigestive anastomoses .Basic problems remains vascularisation of the FAG and adequate epitelisation of the FAG.Protection of the FAG with intraluminaly prothesis and omentoplasty probably decrease stenosis,which is our present experiment.

### V035

INTRAHEPATIC BILIARY DUCT JEJUNOSTOMY ON THIRD SEGMENT (IDDS).

G.De Sena. G.Picardo, F.La Rocca.
Department of Emergency Surgery, "San G.Moscati" Hospital Avellino Italy.

We show our operative technique of intrahepatic jejunostomy on third segment (IDDS) in a case of obstructive jaundice for a common biliary duct carcinoma.
A dilatation of intrahepatic biliary duct is necessary. Before the anastomosis must find the biliary duct of third segment. We use often this operative technique in stead to internal or external drainage. We reserve this traitment for patients without other operative indications in stead to trans hepatic or endoscopic intubation. The best quality of life,even if dont growt the survive, allow the surgical traitment.

### V036

**ENDOSCOPIC INTRACORPOREAL LASER LITHOTRIPSY ( ISWL ) WITH A STONE RECOGNITION PULSED DYE LASER SYSTEM: A NEW APPROACH TO DIFFICULT COMMON BILE DUCT (CBD) STONES.**

F. Schreiber, G. CH. Gurakuqi, M. Trauner and G. J. Krejs.
Internal Department, University Hospital of Karl Franzens University Graz, Austria ( Chairman: G. J. Krejs ).

A consecutive series of 16 patients with difficult common bile duct stones underwent intracorporeal laser lithotripsy with an automatic stone recognition system during a 3 - month period. A pulsed rhodamine 6G laser with a wavelength of 594 nm was used.The integrated stone recognition system shuts off fully energy deliverance when the laser fibre does not reach contact with the stone surface. 15 patients were treated via the peroral route , one patient via the transhepatic route with an ultrathin cholangioscope. All patients ( aged 49 - 89 years, ~ 70 years ) had concrements in the extra - or intrahepatic bile ducts with a mean number of 4 ( 1 - 10 ) and a mean diameter of 22 mm ( 11 - 32 mm). One patient had an inaccessible papilla after gastroduodenal surgery and a CBD stone in front of the strictured pancreatic part of the duct.
Stone disintegration could be achieved in all cases with a mean discharge number of 6800 ( 120 - 25000) and a power setting of 100mJ and within 1, 7 ( 1 - 4 ) sessions on average. The mean session time was 53 min ( 8 - 126 min ). Complete stone clearance could be reached in 14 cases, in two patients small fragments remained in the duct, able to pass spontaneously. In one patient slight hemobilia was seen as a procedure related side effect wich required no special treatment.
In conclusion laser lithotripsy with the stone recognition system is as effective as save in patients with biliary concrements not amenable for conventional endoscopic procedures.

### V037

### JEJUNAL LOOP CHOLECYSTOSTOMY FOR THE TREATMENT OF BYLER'S DISEASE

**T E. Langwieler, X Rogiers, M Malagó, A Sturm*, J Pollock, C. Brunken, M Burdelski*, CE Broelsch**
Department of General Surgery and Department of Pediatrics*, University Hospital Eppendorf, Hamburg, Germany

Byler's Disease is a progressive familial intrahepatic cholestatic syndrome of unclear etiology. It leads to biliary cirrhosis and death before the end of the second decade of life. In our experience this disease represents the second most frequent indication for liver transplantation in children.

The film illustrates a procedure which allows partial diversion of the bile from the enterohepatic bile cycle, thus lowering the bild salt pool. When performed in an early stage of the disease, this operation brings an almost immediate relief of symptoms. In addition it may prolong the course of the disease, bringing these children into an age group where it is easier to find an appropriate organ for transplantation, or even make transplantation unnecessary.

### V038

MODIFIED TECHNIQUE OF THE LICHTENSTEIN'S METHOD FOR SURGICAL CORRECTION OF INGUINAL HERNIA. (PERSONAL MODIFICATION). Papageorgiou E. Department of General Surgery, Pyrgos, General Hospital, Greece.

Over the last few years there has been a spectacular acceleration in the evolution of surgical techniques for correction of inguinal hernia, progression due to the newly obtained knowledge on the biology of hernia as well as the availability of synthetic materials suitable for prothesis and becoming day by day more safe and efficient.

The history of the surgical techniques for correction of inguinal hernia can be devided into two periods: the first characterised by the direct suturing of the hernia and the second one by the use of synthetic prothesis. Today the surgical restoration of inguinal hernia is one of the most common operations all over the world. In the past the operation would require general anaesthesia and the patient would need to stay in the hospital for 6-8 days postoperatively. Modern technology has made the operation so simple that the patient is now required to stay in the hospital for only one day or even go home straight away; it is also possible to perform the operation out of the hospital. The operation is now performed under local anaesthesia and the patient can get up without help from the surgical table and go to bed or even home (however, we keep them in for one day).

The new technique has been made possible thanks to a special mesh (Marlex) combined with a plug (Marlex) which is put deeply into the abdominal inguinal ring if we have to deal with indirect inguinal hernia or into the femoral ring if we have to deal with a femoral hernia. For the direct inguinal hernia we usually use only the mesh and a sort of "cigar or "cigarette" made out of mesh (depending on the size of hernia".

The details of this novel method, which is basically a Lichtenstein's method with a personal modification, are shown in the video.

This is a small contribution to the conviction that a surgeon has to be always aware and in search of new more efficient techniques.

